# Review of Progress in Interventional Therapy for Coronary Bifurcation Lesions

**DOI:** 10.31083/j.rcm2501002

**Published:** 2024-01-04

**Authors:** Chuncheng Gao, Dongdong Li, Huimiao Dai, Hao Liu, Pengyun Liu, Miaomiao Cheng, Mingming Zhang, Wangang Guo

**Affiliations:** ^1^Department of Cardiology, Tangdu Hospital, Air Force Medical University, 710038 Xi’an, Shaanxi, China

**Keywords:** coronary bifurcation lesions, provisional stenting, planned dual-stenting, left main

## Abstract

Despite a decade of extensive research and clinical insights, percutaneous coronary intervention strategies for coronary bifurcation lesions have remained a challenging and highly debated area. This article presents a review of the latest findings and advances in defining and classifying coronary bifurcation lesions, *in vitro* studies, intracoronary imaging, stenting strategies, and the deployment of drug-coated balloons. Based on current evidence, this review provides recommendations for interventional cardiologists to develop individualized interventional strategies and enhance the efficiency of stenting procedures.

## 1. Introduction

Coronary bifurcation lesions (CBLs) constitute 15% to 20% of all percutaneous coronary intervention (PCI) cases [1], and are characterized by significant individual variability in anatomy, complex interventional procedures, perioperative risk, and postoperative complications [2], leading to low success rates of PCI and increased long-term recurrent cardiovascular events. Standardized interventional protocols for all CBLs are impractical given their inherent complexity [3]. Accurate and individualized PCI for CBLs is essential for clinical management. The 17th European Bifurcation Club (EBC) Consensus [4] recommends a stepwise layered provisional stent (PS) implantation as a default strategy for most simple CBLs, with consideration given to a systematic two-stent strategy for a small number of complex CBLs by experienced interventionalists. Moreover, developments in studies involving *in vitro *experiments, simulating CBLs, intracoronary imaging, and drug-eluting stents (DES), have led to improvements in clinical prognosis [5]. This article offers a comprehensive review of the advances in interventional treatment of CBLs developed over the last decade. It aims to provide interventionalists with a concise overview of operational considerations for CBLs, and develop a decision-making flowchart of PCI procedures based on the latest evidence.

## 2. Fundamental Aspects

### 2.1 Definition and Physiological Fractal Geometry of CBL

A CBL is defined as a stenosis in the coronary artery occurring at the beginning of a significant side branch (SB). A significant SB is determined by the operator, taking into consideration various factors such as the patient’s symptoms, concomitant diseases, the internal diameter and length of the SB, plaque burden and location, the angle of the main branch (MB) and SBs, the territory of the myocardium supplied by the SB, and left ventricular function [6].

CBLs consist of a main vessel (MV) and SB. The MV divides into a distal main vessel (dMV) and proximal main vessel (pMV). There is a mismatch phenomenon in that the pMV is larger than the dMV. Theories that have been proposed to explain this phenomenon include Murray’s law, the Area-preservation model, the Huo-Kassab model, and Finet’s law (Table 1) [7]. Finet’s model simplifies the quantitative analysis of coronary bifurcations by focusing on normal angiographic data [8]. This approach has gained widespread use due to its simplicity and effectiveness in evaluating coronary bifurcations [8]. CBL is prone to atherosclerosis due to the unique local blood flow pattern and subsequent endothelial shear stress environment. The side opposite to the carina is particularly vulnerable to atherosclerosis [3]. Factors contributing to poor clinical outcomes post-PCI in bifurcation lesions include disturbed blood flow, areas of low wall shear stress, and vasodilation that deviates from the principles of vessel branching [9, 10, 11]. Martin* et al*. [12] analyzed computational fluid dynamics on the influence of stent and vessel deformation, and demonstrated that stent and vessel deformation are likely to have a major impact on the hemodynamic environment in stented coronary arteries.

**Table 1.** **Common laws of geometric relation between diameters of a bifurcation**.

Model	Geometric relation	Advantage	Limitation
Murray	$D_{\text{pMV}}$^3^ = $D_{\text{dMV}}$^3^ + $D_{\text{SB}}$^3^	Validated in normal and diseased coronary bifurcations by intravascular ultrasonography.	Not applicable to calcified lesion and the culprit lesion of acute coronary syndrome.
		Based on conservation of mass and a minimum energy hypothesis for laminar flow.	Considering wall shear stress is constant throughout the vasculature, which is not supported by experimental measurements.
Area-preservation	$D_{\text{pMV}}$^2^ = $D_{\text{dMV}}$^2^ + $D_{\text{SB}}$^2^	None.	Not supported by vascular anatomical data and experimental observations.
Huo-Kassab	$D_{\text{pMV}}$^7/3^ = $D_{\text{dMV}}$^7/3^ + $D_{\text{SB}}$^7/3^	Based on conservation of mass and a minimum energy hypothesis for laminar flow.	Relatively complex.
Finet	$D_{\text{pMV}}$ = 0.678 ($D_{\text{dMV}}$ + $D_{\text{SB}}$)	Validated for certain branching of human epicardial coronary arteries; simple.	Not applicable to very small $D_{\text{dMV}}$ or $D_{\text{SB}}$.
			Not obey conservation of mass.

$D_{\text{pMV}}$, $D_{\text{dMV}}$, and $D_{\text{SB}}$ represent the diameter of proximal main vessel, distal main vessel and side branch.

Understanding the relationship between different vessel sizes and the scaling relation between the diameter and the myocardial mass perfused are crucial to optimal kissing ballooning, proximal optimization, and SB treatment [13]. A better understanding of the physiologic effects of bifurcation stenting may assist the interventionalist to formulate strategies and create dedicated devices to improve clinical outcomes.

### 2.2 Classification of CBL

The classification of bifurcation lesions can be performed mainly based on the location of plaque distribution and other factors. Common classification systems include the Medina and Lefevre classifications, with the former being widely utilized due to its convenience and accuracy in describing CBL. The Medina classification system is recommended by consensus as the standard classification for bifurcation lesions, with the aim of standardizing and facilitating comparability between relevant findings [14]. The Medina classification system classifies plaque distribution using three points: pMV, dMV, and SB, with 1 or 0 indicating whether the degree of stenosis is greater than 50%. True bifurcation lesions are identified as Medina (1,0,1), (1,1,1), and (0,1,1); the latter two are considered complex bifurcation lesions by default. However, the Medina classification system does not account for risk factors that increase the possibility of SB occlusion, such as lesion length, severity, calcification, thrombosis, bifurcation angle, and SB diameter. Therefore, it is insufficient to develop an interventional strategy based solely on the Medina classification system. Chen* et al*. [15] explored the risk factors for SB occlusion in complex bifurcation lesions and developed the DEFINITION criteria. These criteria identify a complex bifurcation lesion by the presence of at least one major factor combined with two or more minor factors. Conversely, a bifurcation lesion is considered simple if these criteria are not met. DEFINITION criteria offer a more comprehensive and nuanced approach to evaluate risk factors of SB occlusion in CBLs. To classify a CBL as complex according to these criteria, it must exhibit at least one major risk factor, such as stenosis ≥70% and a SB length of ≥10 mm in left main (LM) bifurcation lesions, or stenosis ≥90% and a SB length of ≥10 mm in non-LM bifurcation lesions. Additionally, there are six minor risk factors, including moderate to severe calcification, multiple lesions, a bifurcation angle <45° or >70°, MV reference vessel diameter (RVD) <2.5 mm, thrombus-containing lesions, and MV lesion length ≥25 mm. Based on these criteria, most bifurcation lesions are found to be simple, reaffirming that a stepwise layered provisional stenting approach is the most commonly used procedure for managing most bifurcation lesions.

In addition to the above two commonly used classification criteria, more attention should be paid to Movahed classification [16]. This simplified system employes a combination of letters and numbers to provide a clinically relevant anatomic description of a given coronary artery bifurcation lesion, and includes optional suffixes for any necessary anatomical features of a bifurcation lesion [17]. The Movahed classification is noted for its intuitive, specific, and helpful nature in comparison to the Medina classification, aiding interventionalist in quickly understand lesions characteristics and formulating reasonable interventional strategies.

## 3. Progress of CBL *in Vitro* Experiments

*In vitro* experiments on CBL primarily involve four aspects: bench models, Visible Heart methodologies (*ex vivo* models), computer simulations, and three-dimensional (3D) printing [5]. These methods are utilized to investigate various aspects of CBL, such as stent positioning, deformation, and deployment, as well as flow dynamics and stent performance in response to the different types of bifurcation lesions or stenting strategies [18, 19, 20, 21].

The use of *in vitro* models is predominantly centered around the creation of bifurcation vessel models using various materials such as metal, glass, aliphatic polyether-based thermoplastic polyurethane, and silicone. The ideal material allows transparency to observe or photograph the model, assess elasticity, and anatomical accuracy to simulate blood vessels. Among these, the silicone *in vitro* model recommended by the EBC [22] stands out as the most extensively utilized and beneficial option in experimental studies pertaining to CBLs. The EBC provides data on required size, angle, and vascular elasticity parameters for model construction, promoting the development of* in vitro* experiments [23, 24, 25].

Silicone models have many advantages, including simplicity, convenience, and accessibility. They offer the capability to evaluate various stent parameters in conjunction with internal cavity imaging and scanning electron microscopy (EM). Additionally, they serve as valuable tools in demonstrating interventional techniques for teaching purposes. Nevertheless, it is crucial to acknowledge the limitations of silicone models. They fall short in accurately capturing the complexity of human coronary arteries, and fail to represent the dynamic changes in bifurcations that occur throughout the cardiac cycle.

An *ex vivo* model employs specimens from pig or cadaveric human hearts, facilitating the simulation of interventional procedures under X-ray guidance within an extracorporeal circulation device. This model is commonly used to assess interventional techniques and evaluate the effect of stent strategies using internal cavity imaging [26]. The advantage of an *ex vivo* model is the ability to replicate the coronary vascular elasticity and anatomy, enhancing the realism of the simulation and providing more valuable reference and guidance for actual clinical procedures [27, 28]. Despite these advantages, there are notable challenges associated with ex vivo models. Obtaining the necessary experimental materials can be difficult, the complexity of the required operational procedures is high, and there is an imperative need for stringent ethical approval to conduct experiments using these models.

Computer simulation models utilize specialized software to recreate a surgical operation without the need for physical objects. The software is programmed to operate with pre-set parameters, yielding results similar to those from real *in vitro* experiments [29, 30]. Mortier* et al*. [31] evaluated the effects of two final kissing balloon inflation (KBI) strategies by conducting finite element computer simulations of virtual deployment and post-expansion. Using the stent parameters obtained from real *in vitro* model tests, they verified the accuracy of the virtual experiments. Computer simulation models generate reliable and stable data without the need for *in-vivo* models, thus saving experimental costs and enable testing a wider range of scenarios [31]. Computer simulation models can efficiently simulate lesion anatomy, plaque size, stent, balloon, and material properties, allowing for the calculation of fluid dynamics and solid mechanical features and producing accurate results.

3D printing of cardiac blood vessels is an emerging technology that utilizes high-resolution bioprinters and various bioinks to construct cardiovascular tissues with complex hierarchical structures with mechanical and biological activity [32]. The printing of arterial systems with real anatomical structures and functions can overcome the limitations of partial silicone *in vitro* models and advance the development of *in vitro* trials for coronary bifurcation therapy. However, this technology is currently limited by the performance of bioinks and is still in the initial stage of development [33].

## 4. Quantitative Coronary Angiography—Dedicated Systems and Software for Bifurcations

Quantitative Coronary Angiography (QCA) is an objective and scientific method to assess the vessel diameter/lesion length and degree of lumen stenosis [34]. However, the application of single-vessel 2D-QCA analysis has been shown to be inaccurate for the assessment of bifurcation lesion dimensions [35]. To accurately quantify lesion severity in CBLs, the use of dedicated bifurcation software is imperative with 2D-QCA [36, 37]. Studies have shown that the dedicated bifurcation-QCA packages (Table 2) outperforms both operator experience using visual inspection methods and conventional 2D-QCA in terms of accuracy and reproducibility [38]. However, 2D- and dedicated bifurcation-QCA were based on a single angiographic projection to estimate lesion geometry and length, as well as the assessment of circular lumen cross sections, leading to coronary vessel overlapping, tortuosity and shortening. 3D-QCA packages have been developed to overcome this shortcoming combining information from two angiographic projections with extract 3D lumen contours [39]. Previous studies have shown that 3D-QCA is superior to 2D-QCA in predicting reduced fractional flow reserve (FFR) and assessing functional stenosis [40, 41].

**Table 2.** **Main studies assessing efficacy of 3D-QCA-based software in assessing clinical effects**.

Study title/Start year	Software	Objectives	Design	Patients	Publications
ReVEAL iFR	Angio iFR	To evaluate the diagnostic accuracy of the Angio iFR software in estimating iFR and FFR from 3D-QCA reconstructions	Prospective observational	650	Available
2019					(NCT03857503)
QIMERA-I	QFR	To assess the accuracy of QFR estimated following virtual angioplasty against invasive physiological indices and true QFR measured post-PCI	Prospective observational	100	Unavailable
2020					(NCT04200469)
QFR-STEMI	QFR	To compare the clinical effects of QFR-guided with angiography-guided revascularization on non-culprit vessel in STEMI patients with multi-vessel lesions	Prospective Double-blind RCT	6800	Unavailable
2020					(NCT04259853)
FAVOR III China	QFR	To compare outcomes between angiography- and QFR-guided PCI	Prospective Double-blind RCT	3860	Available
2018					(NCT03656848)
FAVOR III EJ	QFR	To compare outcomes between angiography- and QFR-guided PCI (Non inferiority study)	Prospective Single-blind RCT	2000	Unavailable
2018					(NCT03729739)
Flash FFR II	caFFR	To compare outcomes between FFR- and caFFR-guided PCI (Non inferiority study)	Prospective Single-blind RCT	2132	Unavailable
2021					(NCT04575207)

iFR, instantaneous wave-free ratio; QFR, quantitative flow reserve; caFFR, coronary angiography–derived fractional flow reserve; PCI, percutaneous coronary intervention; 3D-QCA, three-dimensional-quantitative coronary angiography; STEMI, st-segment elevation myocardial infarction; RCT, randomized controlled trial.

Recent advancements in 3D bifurcation QCA has been found to calculate optimal viewing angles of bifurcation lesions and further enhance the accuracy of quantitative assessment including bifurcation angle and lesion length. In addition, several types of software have been developed to assess the functional component of the CBL from 3D-QCA without any invasive physiology measurements or the induction of hyperemia [42, 43]. Some software has been validated using *in vitro* models and clinical studies. In a recent study, “ReVEAL iFR”, Angio-iFR was demonstrated to enable operators to accurately predict both the instantaneous wave-free ratio (iFR) and FFR value within a few seconds from a single projection of cine angiography. In another study, “FAVOR III China” found that a quantitative flow ratio (QFR)-guided strategy of lesion selection improved 1-year clinical results in patients undergoing PCI, compared with standard angiography guidance. However, evidence that dedicated QCA systems and Software-guided PCI improves clinical outcomes are still lacking (Table 2) [44, 45].

## 5. Progress of CBL in Intracoronary Imaging

Intracoronary imaging offers significant advantages over traditional coronary angiography for evaluating complex bifurcation lesions, SBs, lesion coverage, guiding wire location, stent expansion, and stent location. It is a crucial tool for balloon delivery and optimal stent implantation in CBL interventions [46]. Intravascular ultrasound (IVUS) and optical coherence tomography (OCT) are the two most commonly used methods.

IVUS has established itself as a crucial imaging technique in the selection and optimization of stent strategies for LM bifurcation lesions and chronic total occlusion (CTO). Despite its widespread use, the traditional IVUS is often critiqued for its lower resolution and somewhat inconsistent image quality when compared to OCT. To bridge this gap, advancements have led to the development of high-resolution (HR) IVUS systems. These systems boast superior image clarity, expedited imaging capabilities, and refined operating elements, thus greatly improving the efficiency of PCI. HR-IVUS precisely evaluates the entire vessel wall with higher near-field resolution and tissue penetration, and retains the potential advantage of conventional IVUS over OCT imaging [47]. Previous studies have shown that IVUS systematically overestimates lumen area compared to OCT, due to low resolution. The application of HR-IVUS can eliminate these differences [48]. Garcia-Guimaraes* et al*. [49] found that HR-IVUS was better at displaying the extravascular elastic membrane compared with traditional IVUS. Although HR-IVUS’s requirement for contrast injection risks inducing or aggravating coronary dissection, it has become an attractive alternative [49]. Nonetheless, there are currently limited reports on the clinical application of HR-IVUS, and further studies are needed to investigate its impact on CBL stenting.

The tissue penetration of OCT is low (only 1–3 um), and contrast injection may induce or aggravate coronary dissection. In addition, OCT cannot evaluate larger diameter vessels [50]. Real-time 3D OCT allows interventionalists to analyze CBLs from all angles, leading to a more accurate assessment of branch guiding wire reentry position [51]. In a study investigating the feasibility and effectiveness of 3D OCT-guided optimal lateral therapy in CBL stenting, it was found that this technique provides a better view of the SB port and uses a different color-coded stent column to guide the wire into the appropriate mesh [52]. Additionally, the OCTOBER trial has demonstrated that the incidence of major adverse cardiac events (MACE) at 2 years was significantly lower in the OCT-guided group than in the angiography-guided group (10.1% vs. 14.1%; HR 0.70, 95% CI 0.50–0.98; *p* = 0.035), indicating that OCT guidance for complex bifurcation lesions is safer than conventional coronary angiography [53]. However, the study’s design has some limitations compared to real world practice. The number of LM bifurcation lesions in the experiment was small, which reduced the risk in the overall population. More evidence from well-designed randomized controlled trials (RCTs) are needed.

## 6. Stenting Strategy for CBL

The 17th EBC consensus on CBL recommends the use of a stepwise layered PS for simple CBLs, while a planned two-stent strategy is recommended for complex lesions. The decision to proceed with a two-stent technique is heavily influenced by the anticipated complexity of rewiring the SB following-stent placement [5]. In cases where the angle between the distal MV and SB is too large, or the SB opening is too twisted, alternative options for SB occlusion after MV stenting must be considered. In such cases, blood flow to the SB can be compromised following MV stenting, leading to the failure of rescue guidewire maneuvers and rescue two-stent implantation. In this case, a planned two-stent operation would be a reasonable approach. Additionally, drug-coated balloons (DCB) represent a new option for SB therapy, which require lesion pretreatment. Therefore, a planned double stenting or a provisional stenting approach may also be an option. To avoid the need for double stent implantation, a “fine pretreatment” approach may be used to gradually treat the SB prior to stenting.

**Fig. 1.**
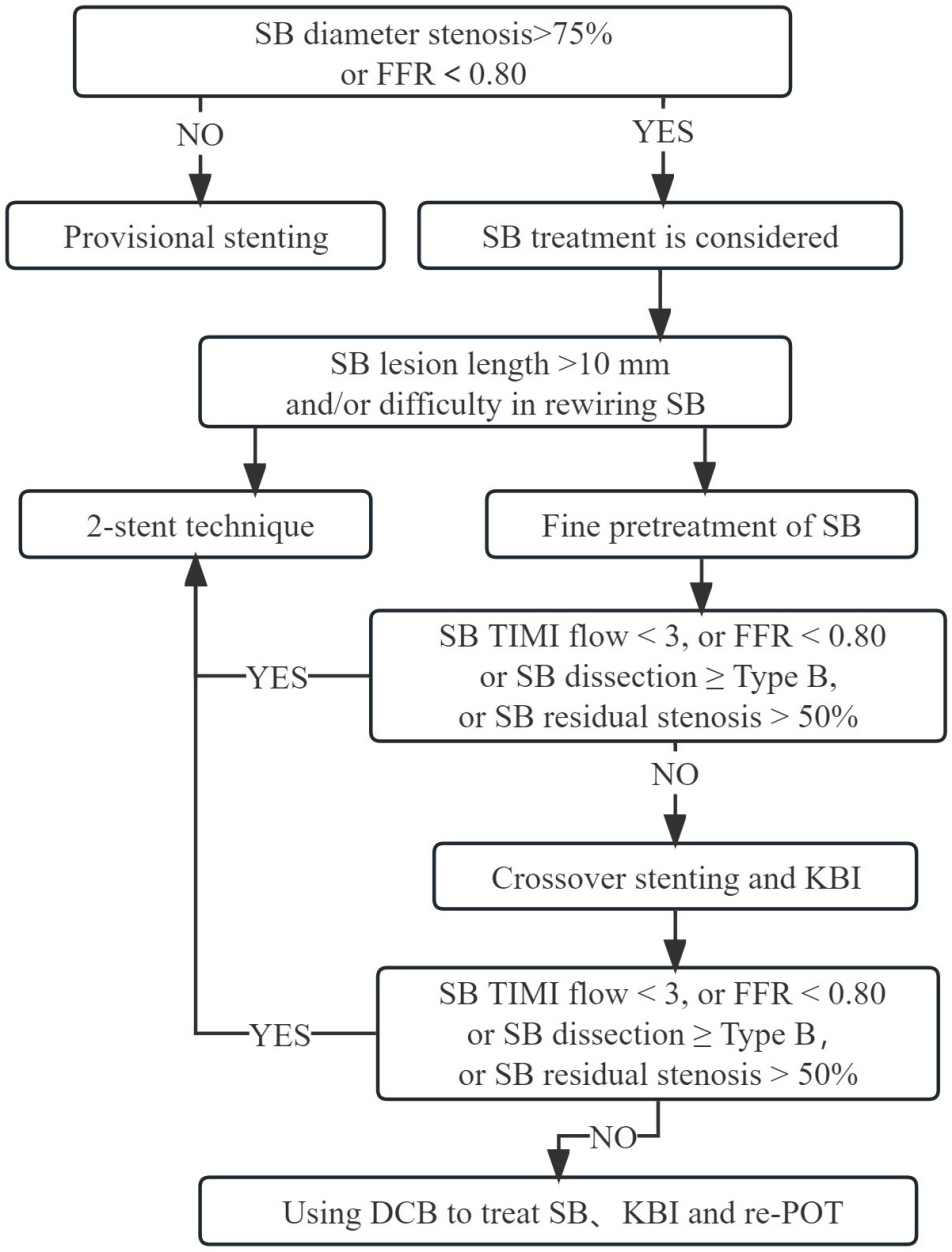
**Strategies for coronary bifurcation lesions intervention**. SB, side branch; FFR, fractional flow reserve; KBI, kissing balloon inflation; TIMI, thrombolysis in myocardial infarction; DCB, drug-coated balloon; re-POT, repeat proximal optimization technique.

A possible strategy for selecting the appropriate treatment is to first expand a small balloon with low pressure for an extended period of time, followed by expanding a cutting balloon. According to the results of the pretreatment, a decision can be made to either directly implant the DCB or a planned two-stent approach (Fig. 1), A SB needing significant protection is characterized as having a diameter ≥2.0 mm. Excessive protective measures should not be considered when the SB diameter is <1.5 mm; The “keep it open” (KIO) principle was implemented when the SB diameter is ≥1.5 mm and <2.0 mm. A double-stent strategy should be considered when the SB diameter is >2.75 mm and the lesion is located at the ostium to the proximal middle segment. Furthermore, the length of the SB is another factor to be considered [54]. In addition, the location of the guidewire rewiring is also the key to determine the intervention strategy for CBLs. *In vitro* experiments have demonstrated that recrossing the distal stent cell, as opposed to the proximal cell, results in a larger opening area of the SB, as well as a lower rate of the mal-apposition of the stent breaking into the SB, significantly influencing the success of the procedure [55].

In the double-stent strategy, the location of the rewiring is particularly important. It not only influences the selection of the two-stent technique but also bears risks for suboptimal stent coverage, potential stent distortion, and in severe cases, may lead to irreparable procedural failure [14, 56]. Furthermore, different type of stents may affect the short- and long-term results in PCI for bifurcated lesions. Recently, Choi* et al*. [57] demonstrated that the use of second-generation DES is effective in reducing target-lesion failure compared with first-generation DES. A recent meta-analysis demonstrated that DCBs may be an excellent treatment option for the SB lesions in coronary bifurcations [58].

Bioresorbable scaffolds (BRS) are recommended by EBC in T-stenting for CBLs with 2 BRS or 1 BRS in the main branch and 1 DES in the SB [59]; Choosing a stent from appropriate model designs may lead to optimal stenting results for SB, particularly in the treatment of large bifurcation lesions [60, 61]. Many factors need to be considered to decide on an appropriate CBL treatment strategy. Therefore, what is the choice between PS and dual stent strategy for CBLs? The DEFINITION II Study reported that excessive PS may lead to increased MACE [62], while the EBC MAIN suggested that PS may achieve satisfactory outcomes [63]. Therefore, a comprehensive preoperative and intraoperative evaluation is necessary to make dynamic treatment decisions according to the characteristics of the lesions and pretreatment results, in order to provide individualized strategies.

### 6.1 Key Techniques to Improve the Clinical Outcomes after CBL Stent Implantation

#### 6.1.1 Kissing Balloon Inflation Technique

KBI is a dual balloon manipulation technique unique to bifurcation lesion PCI, designed to benefit both MV and SB in PCI. The purposes of KBI are to reshape the ridge and polygonal area, to restore the shear stress and blood flow velocity of the lateral wall of the bifurcation, and to open the SB. However, KBI may also result in poor adherence, elliptical deformation, and excessive expansion of the proximal part of the MV stent, which might in turn lead to a higher restenosis rate in the MV and increase the risk of SB opening and dissection [64]. Therefore, non-compliant (NC) balloons are often used for balloon inflation, and proximal optimization technique (POT) is commonly employed to enhance the proximal part of the MV stent after KBI. The COBIS registry revealed that KBI in single stent implantation only improved the restenosis of SB, with no increase in the incidence of MACE, and even increased the incidence of overexpansion of stents in proximal vessels and the revascularization rate of MV. For the double stenting technique, KBI can significantly decrease the rate of target lesion revascularization (TLR) and MACE. A recent large registry study revealed that short overlapping KBI was associated with lower restenosis rates in patients with bifurcation or unprotected LM coronary lesions implanted with ultrathin stents. In a dual-stent strategy, KBI was also associated with less TLR and restenosis [65]. Generally, the efficacy of KBI with a single crossover stent is still unclear; however, it is mandatory for double stenting.

A modified KBI procedure involves overlapping and aligning the minimum proximal parts of the two NC balloons above the bifurcation crest, followed by synchronized KBI. Alternatively, the SB balloon could be first inflated and then deflated, followed by MV balloon dilation and finally KBI [66]. Studies on balloon inflation duration have suggested that a balloon inflation time of no less than 25 seconds is ideal for full stent expansion [67]. However, in clinical practice, it is important to take into account the specific intraoperative situation. Asynchronous decompression can potentially cause deviation of the bifurcation ridge, leading to worse clinical results.

#### 6.1.2 Proximal Optimization Technique

It has been proposed that POT can improve the long-term prognosis of CBL intervention. Following stent expansion, a NC balloon with the same diameter as the pMV is used to re-expand the stent near the bifurcation ridge, enabling the stent to conform to the morphology of the bifurcation vessels and optimize the adherence, expansion, and morphology of the proximal segment of the stent [14]. Intracoronary imaging studies have demonstrated that stent endothelialization is delayed in bifurcation lesions without POT, which can lead to stent thrombosis and stent-based restenosis. Finet *et al*. [68] used a bifurcation bench model to compare 6 optimization sequences for CBLs PS, and the results showed that the re-POT (initial POT+SB inflation + final POT) significantly optimized the final result of PS, resulting in better circular geometry while significantly enhancing the ostium area of the SB scaffold and reducing proximal area overstretch and strut mal-apposition, therefore, re-POT may be more effective than optimization techniques related to KBI. Dérimay* et al*. [69] demonstrated that final POT fails to completely correct all proximal elliptic deformation associated with KBI or its derived techniques. The e-ULTIMASTER study has demonstrated that POT can effectively reduce the incidence of TLF at one year, from 6.0% to 4.0% (*p* = 0.01) [70]. As a result, POT is recommended in the EBC expert consensus, and is also often used to significantly decrease proximal stent elliptical changes after MV stent implantation and following KBI procedures. Re-POT maybe even more promising.

During POT, the balloon should be positioned so that the distal shoulder is just at the carina cut plane [71]. Insufficient balloon placement may result in inadequate stent expansion, while over-placement could also cause excessive dilation of the distal MV, leading to vessel dissection, ridge displacement and even perforation. This may require a series of remedial procedures, such as mesh exchange of the guidewire, SB dilation, re-POT, or SB stent implantation. Additionally, the additional 6–10 mm of length (minimum length of common POT balloons) used during the procedure should be considered when selecting the length of the MV stent. The diameter ratio between the balloon and the proximal MV reference segment should be 1:1, and a NC balloon should be used [72]. However, in practice, the accuracy of balloon positioning during POT is also dependent on the operator’s experience, balloon design, and the best selection of angiographic projection. Therefore, POT can be another significant factor that contributes to blood flow damage in SB. As such, the EBC expert consensus suggests protecting the SB before POT. Currently, balloons with short shoulders have been designed for accurate positioning by the Brosmed Medical Company from China. A related multicenter randomized controlled study (NCT05368129) is ongoing. However, multiple projections are still required to obtain satisfactory positioning of the POT balloon.

It is important that POT should be applied at least twice in the double stenting procedure. The initial instances of POT serve to reposition the proximal struts and open the strut cell of the SB ostium, which might decrease the incidence of wire misplacement. The distal ridge should be placed close to the level of the carina to avoid this complication. While the final POT is used to decrease proximal oval deformation, the balloon should only be placed in the pMV segment.

### 6.2 The Main Side Branch Protection Technique of Provisional Stenting 

#### 6.2.1 Jailed Wire Technique

The jailed wire technique (JWT) is a procedure that involves implanting a stent in the MV, while simultaneously “jailing” a guidewire in the SB. This technique has been recommended by the EBC expert consensus as a routine SB protection strategy [73]. However, recent research has cast doubt on the necessity of systematically applying JWT across all cases treated with the 1-stent strategy, suggesting its recommendation should be limited to true CBLs with severe stenosis of the SB or MV. The incidence of final SB occlusion after MV stenting was significantly lower in the JWT group than in the non–JWT group, and the long-term clinical results of two groups were comparable [74]. Although the jailed wire may not fully keep the SB open, it can be used as a pathway for the guidewire to re-enter the SB through the strut cells in case of occlusion. In extreme cases, it can be employed as a rescue pathway to restore blood flow to the SB by advancing a small balloon underneath the stent [75]. The application of JWT requires consideration of various factors, including the degree of calcification, the length of the trapped wire, and the deployment pressure of the MV stent, which may lead to failure of the JWT such as wire fracture or difficulty with retraction. A recent study reported that polymer-coated wires appear to be more resistant to damage during wire retraction than non-polymer-coated wires, thus reducing the risks of wire breakage and retraction failure [76].

#### 6.2.2 Jailed Balloon Technique

**Fig. 2.**
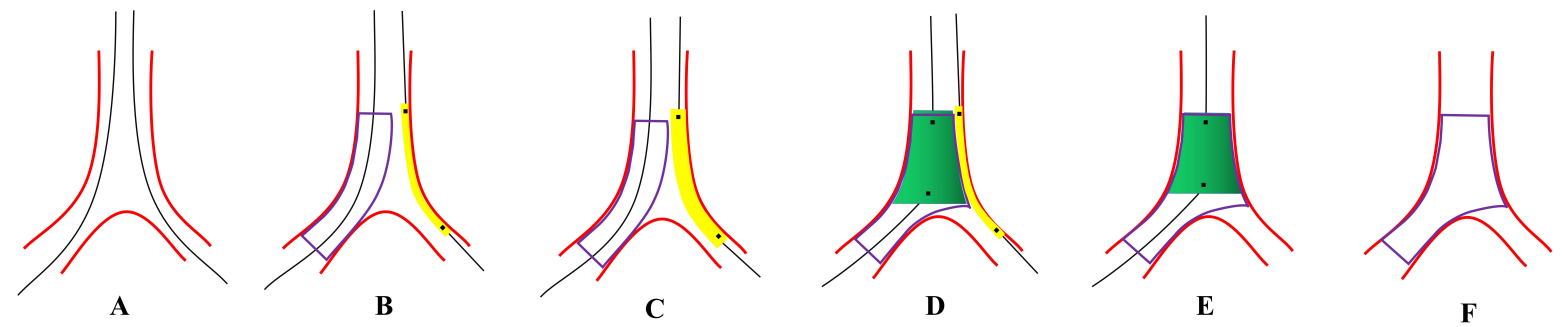
**Recommended steps of jailed balloon proximal optimization technique (JB-POT)**. (A) Both the main vessel (MV) and side branch (SB) are wired. (B) The main vessel stent is then deployed with a balloon jailed in the SB. (C) The jailed balloon is dilated at 6–8 atm if SB blood flow is degraded. (D) POT and post-dilation of the distal stent were performed with non-compliant (NC) balloons of corresponding sizes. (E) The jailed balloon is retrieved and repeat-POT is performed 2 mm away from the SB branching point. (F) Final effects are examined.

The jailed balloon technique (JBT) overcomes the limitations of JWT and has shown superior immediate procedural success rates with excellent SB protection over JWT for complex, true bifurcation lesions. It was first mentioned by the 17th EBC expert consensus as a SB protection strategy [4]. Two recent registry studies demonstrated that JBT results in a significant reduction in the incidence of SB occlusion in comparison with JWT [77, 78]. The CIT-RESOLVE trial randomized 335 patients at high risk for SB occlusion to an active strategy group (JBT for small SBs) or a conventional strategy group (JWT for small SBs). The study showed that the active SB protection strategy was superior to the conventional strategy and was associated with a significantly lower incidence of SB occlusion and SB blood flow loss immediately after the MV stent was fully attached. However, subgroup analyses showed no significant differences in major adverse cardiac events (MACE) at 1-year follow-up [79]. Despite these promising results, EBC has not included JBT in their preferred recommendations, perhaps due to the lack of large-scale RCT studies on JBT and JWT and their derivative techniques, and considering that JWT is easier to perform. However, in clinical practice, JBT has demonstrated effectiveness in reducing the incidence of border branch occlusion and is considered a safe alternative. The Jailed Balloon Proximal Optimization Technique (JB-POT), is a novel approach that combines JBT and POT created by our team [80]. Fig. 2 illustrates the operation steps involved in JB-POT, which aims to simplify the process of PS by combining JBT and POT. Ongoing multicenter studies are currently being conducted to assess the procedure.

#### 6.2.3 Side Branch Pre-Dilation Technique

SB pre-dilation involves balloon dilation before the MV stent implantation. Evidence suggests pre-dilation has several benefits, including increasing SB ostial area to facilitate SB stenting, improving SB blood flow, and reducing excess SB intervention [81, 82]. But based on current studies, routine pre-dilation of the SB is not recommended. Studies have identified the increased risk of SB dissection and subsequent challenges in rewiring the SB, ultimately resulting in adverse events [83]. However, it can be used when severe stenosis or angular lesions exist in the SB ostium resulting in impaired blood flow after the guidewire enters the SB.

### 6.3 Bail-Out Two-Stent Technique Selection of Provisional Stenting Approach for CBL

**Fig. 3.**
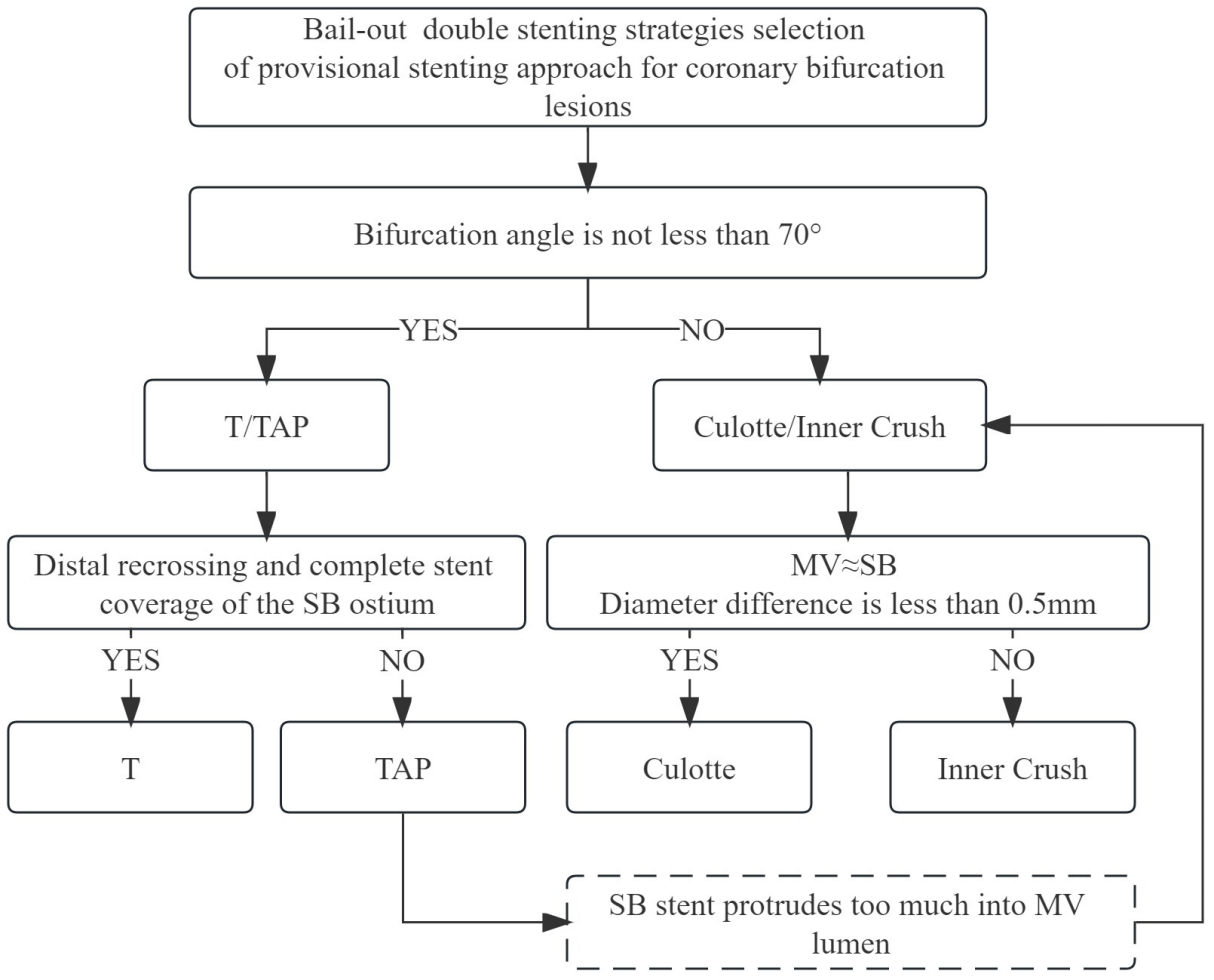
**Bail-out two stenting selection of provisional stenting approach for coronary bifurcation lesions**. T, T-stenting technique; TAP, T-stenting and the small protrusion technique; SB, side branch; MV, main vessel.

In the PS strategy, there is a variance in the necessity for a second stent, ranging from 1–41% of cases [84, 85]. The need for Remedial stenting in SB typically arises under specific circumstances as follows: the Thrombolysis in Myocardial Infarction (TIMI) flow is <grade 3, the SB has ≥type B dissection, the fractional flow reserve (FFR) is <0.8; and the residual SB stenosis is >50% following balloon dilation [86, 87]. To optimize the outcome of PS, the following should be considered: the guidewire should be inserted through the distal strut cell, and the stent should enter the SB after dilation. To enhance the chances of crossing the distal strut cell, a tangential view of the SB should be maintained. OCT imaging can assist in accurately assessing the position of the crossing wire and its relationship to the stent. Procedures involving two-stent techniques include T-stenting, T-stenting and the small protrusion technique (TAP), culotte, and inner crush techniques. The specific strategy (Fig. 3) is determined by the angle of the bifurcation lesion, the position of the SB guidewire through the stent strut, and the difference in diameter between the MV and SB.

#### 6.3.1 Provisional T and TAP Stenting

**Fig. 4.**
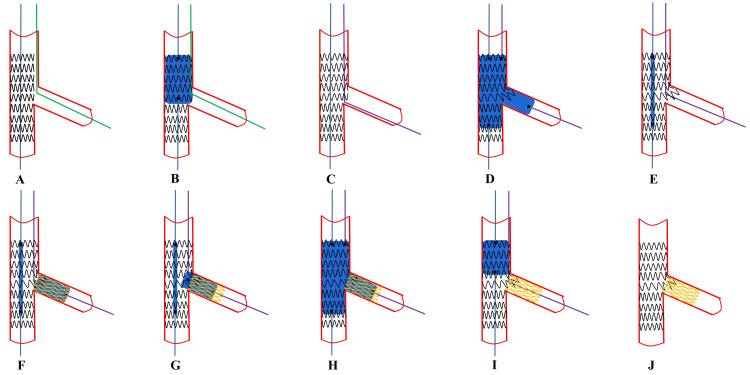
** 
Recommended steps of provisional T stenting**. (A) MV stent is deployed with a jailed wire in the SB. (B) Initial POT. (C) Distal SB rewiring according to the pullback technique and a second guidewire is placed in the MV. (D) Simultaneous KBI with MV balloon sized 1:1 according to distal MV and SB balloon sized 1:1 according to SB diameter. (E) Placement of a NC balloon into the MV sized 1:1 according to the distal MV diameter. (F) Select the best position of the SB stent to connect struts of the MV protruding into the SB and fully cover SB lesion. (G) After SB stent deployment, the balloon of the stent is slightly pulled back and repeated inflation at high pressure is performed in order to warrant optimal stent expansion at the level of SB ostium. (H) After alignment of the MV balloon and SB non-compliant balloon, kissing balloon inflation is performed by inflating simultaneously these two balloons. (I) A repeat proximal optimization technique is considered. (J) Final effects are examined. SB, side branch; MV, main vessel; NC, non-compliant; POT, proximal optimization technique; KBI, kissing balloon inflation.

**Fig. 5.**
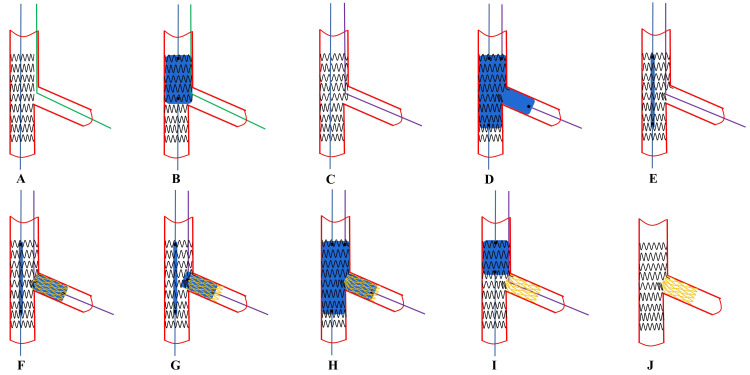
**Recommended steps of provisional TAP stenting**. (A,B) These steps are the same as for T-stenting. (C) SB rewiring according to the pullback technique and a second guidewire is placed in the MV. (D,E) These steps are the same as for T-stenting. (F) After MV stent deployment, the SB stent is precisely positioned to just fully cover the upper edge of the SB ostium, while the stent at the distal edge of the SB ostium protruded slightly into the MV about 1–2 mm, the stent is inflated while the MV balloon is kept un-inflated. (G–J) These steps are the same as for T-stenting. TAP, T-stenting and the small protrusion technique; SB, side branch; MV, main vessel.

The T or TAP stent technique is preferred when the angle between MV and SB is ≥70°. TAP is a modified T-stent technique that involves precisely positioning the SB stent to cover only the proximal edge of the branch opening after LM stent placement [88]. However, the stent at the distal edge of the SB ostium protrudes slightly into the LM by approximately 1–2 mm to ensure full coverage of the SB ostium. This technique can minimize overlap of the multiple scaffold layers at the bifurcation site. If the guidewire of the SB re-entrant has successfully passed through the distal cell of SB, and the stent strut of SB has attached to the upper edge via anastomosis expansion, a T-stent can be obtained (Fig. 4). Otherwise, a TAP operation is required to ensure proper coverage (Fig. 5). Accurate positioning of stent deployment is critical in achieving optimal T or TAP stent placement. An excessive deep deployment of the SB stent can result in inadequate stent coverage of the SB ostium, increasing procedural complexity and the incidence of MACE [89]. The KBI after SB placement of TAP stent creates a new metal carina above the original one. The length of the metal carina is primarily influenced by the length of the SB stent protrusion into the MV, while its morphology is mainly determined by the quality of the KBI. The length and bias of the carina affects vascular endothelialization and the occurrence of MACE. Therefore, it is necessary to standardize the KBI procedure and control the length of the SB stent to ensure that the metal carina is aligned with the median line and the length of the MV is optimized. If the SB stent protrudes too far into the MV, the interventional technique should be switched from TAP to the culotte or crush protocol.

#### 6.3.2 Provisional Culotte and Inner Crush Stenting

**Fig. 6.**
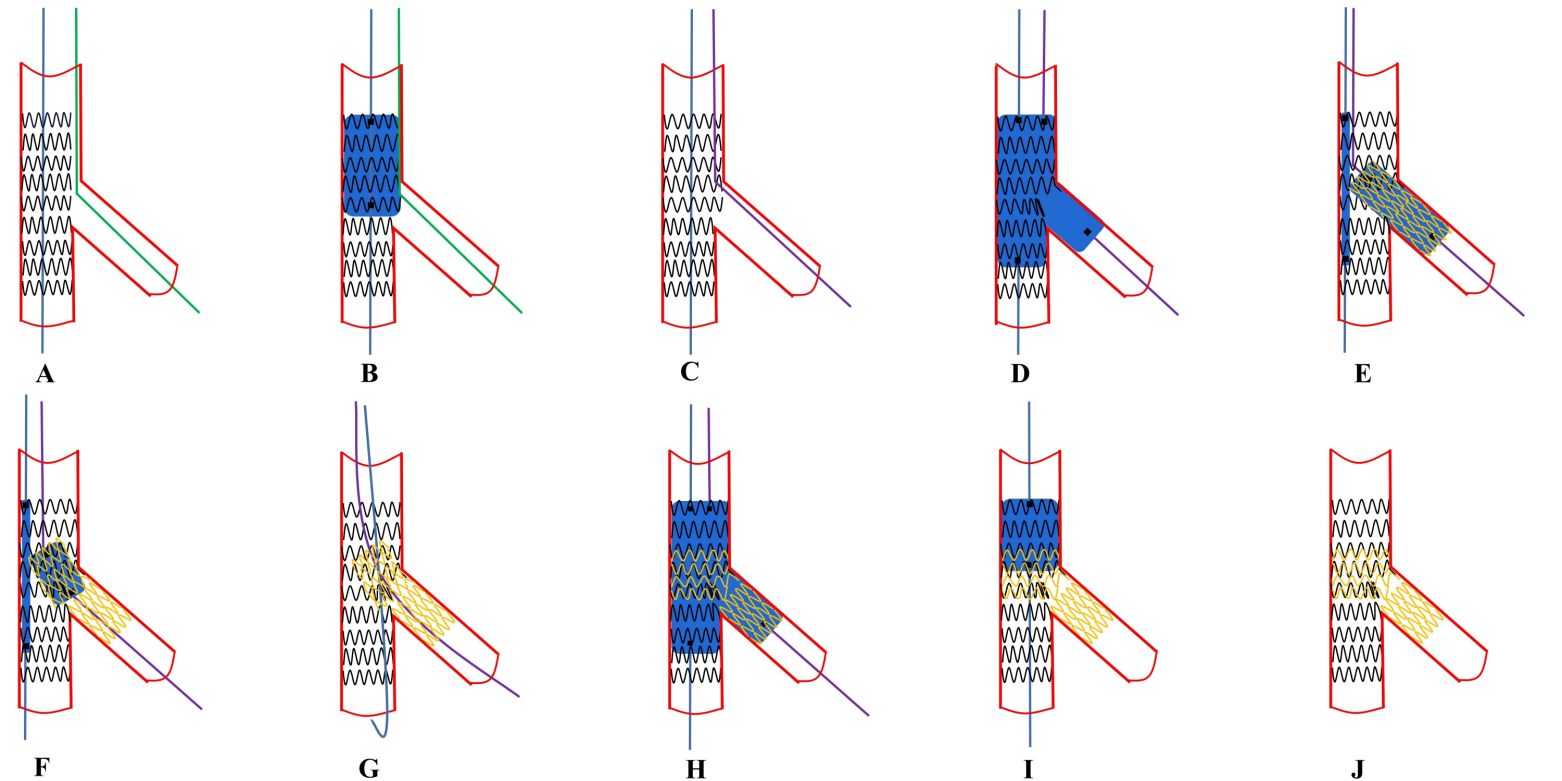
**Recommended steps of provisional Culotte stenting**. (A,B) These steps are the same as for T-stenting. (C) SB proximal rewiring according to the pullback technique and a second guidewire is placed in the MV. (D) The step is the same as for T-stenting. (E) A SB stent implantation across the first MV stent with a diameter selected 1:1 according to the SB size, and a length selected to ensure SB lesion coverage, with a balloon kept un-inflated in MV. (F) Complete POT with a balloon diameter sized 1:1 according to the proximal MV, with a balloon kept un-inflated in MV. (G) Distal rewiring of the first stent with SB guidewire according to the pullback technique and a second guidewire enters the MV stent, then SB stent, and finally to the distal end of SB with the knuckle guidewire technique. (H) KBI is systematically performed. (I) A repeat POT is considered to avoid neocarina. (J) Final effects are examined. SB, side branch; MV, main vessel; POT, proximal optimization technique; KBI, kissing balloon inflation.

**Fig. 7.**
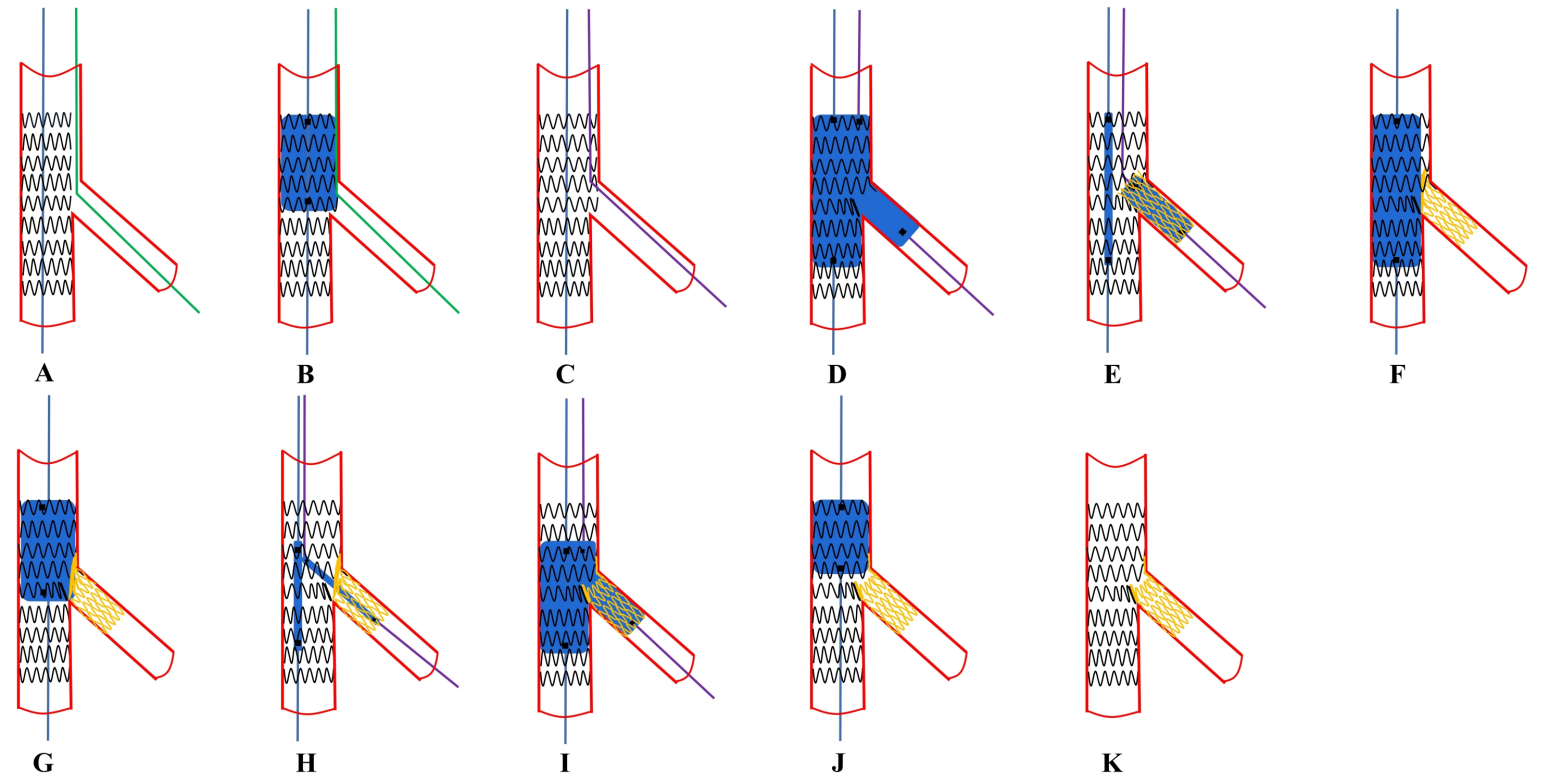
**Recommended steps of provisional inner Crush stenting**. (A,B) These steps are the same as for T-stenting. (C) SB proximal rewiring according to the pullback technique and a second guidewire is placed in the MV. (D) The step is the same as for T-stenting. (E) SB stenting. While the MV balloon is kept uninflated in the MV, the stent (sized 1:1 according to SB) is implanted in the SB protruding into the proximal MV by 2–3 mm. (F) Balloon crush. After removal of the SB stent’s balloon, the protruding struts of the SB are crushed by the inflation of the balloon inside the MV sized to the distal MV. This initial balloon crush is theoretically incomplete resulting in stent malapposition in the proximal MV (pMV). (G) POT crush with a short balloon sized 1:1 to the pMV to warrant optimal crushing without pMV stent malapposition. (H,I) Proximal or non-distal SB rewiring and KBI using two non-compliant balloons sized 1:1 according to the SB and distal MV diameters. (J) A repeat POT is considered to avoid neocarina. (K) Final effects are examined. SB, side branch; MV, main vessel; POT, proximal optimization technique; KBI, kissing balloon inflation.

Culotte stenting can be an optimal choice when the angle between the MV and SB is <70°, or when there is an excessive protrusion of the SB stent into the MV stent and the diameter difference between the SB and MV is less than 0.5 mm [56]. A guidewire passes through the proximal strut cell into the SB to complete the KBI. After the SB stent is implanted, KBI is completed followed by repeat POT [90]. One of the critical issues encountered in Culotte stenting is the formation of a “waist sign”. To circumvent this complication, it is imperative to strictly observe the limits of the differences between the main and side support diameters and selecting a support platform with a sufficiently large support unit ring. The expansion of the SB stent may also prompt the closure of the MV. To prevent this issue, the “jailed balloon in the MV” technique can be employed, as shown in Fig. 6. When the diameter difference between the MV and SB is >0.5 mm, the inner crush stent can be used. The SB stent is pressed against the side wall of the MV, and then the guide wire is passed through the non-distal cell strut into the SB to complete the KBI and repeat POT. The inner crush stenting technique often presents technical difficulties due to the accumulation of two or three layers of stents near the SB ostium. This makes it challenging to rewire the SB and subsequently insert the balloon. Common approaches to address this challenge include: parallel insertion and pulling back the guidewire, replacing the guidewire with a stiffer one of a different size, shaping the guidewire’s tip to match the SB characteristics, providing support and pushing direction for the guidewire through the microcatheter, and optimizing the SB ostium. The specific steps are shown in Fig. 7.

### 6.4 Planned Two-Stent Strategy

**Fig. 8.**
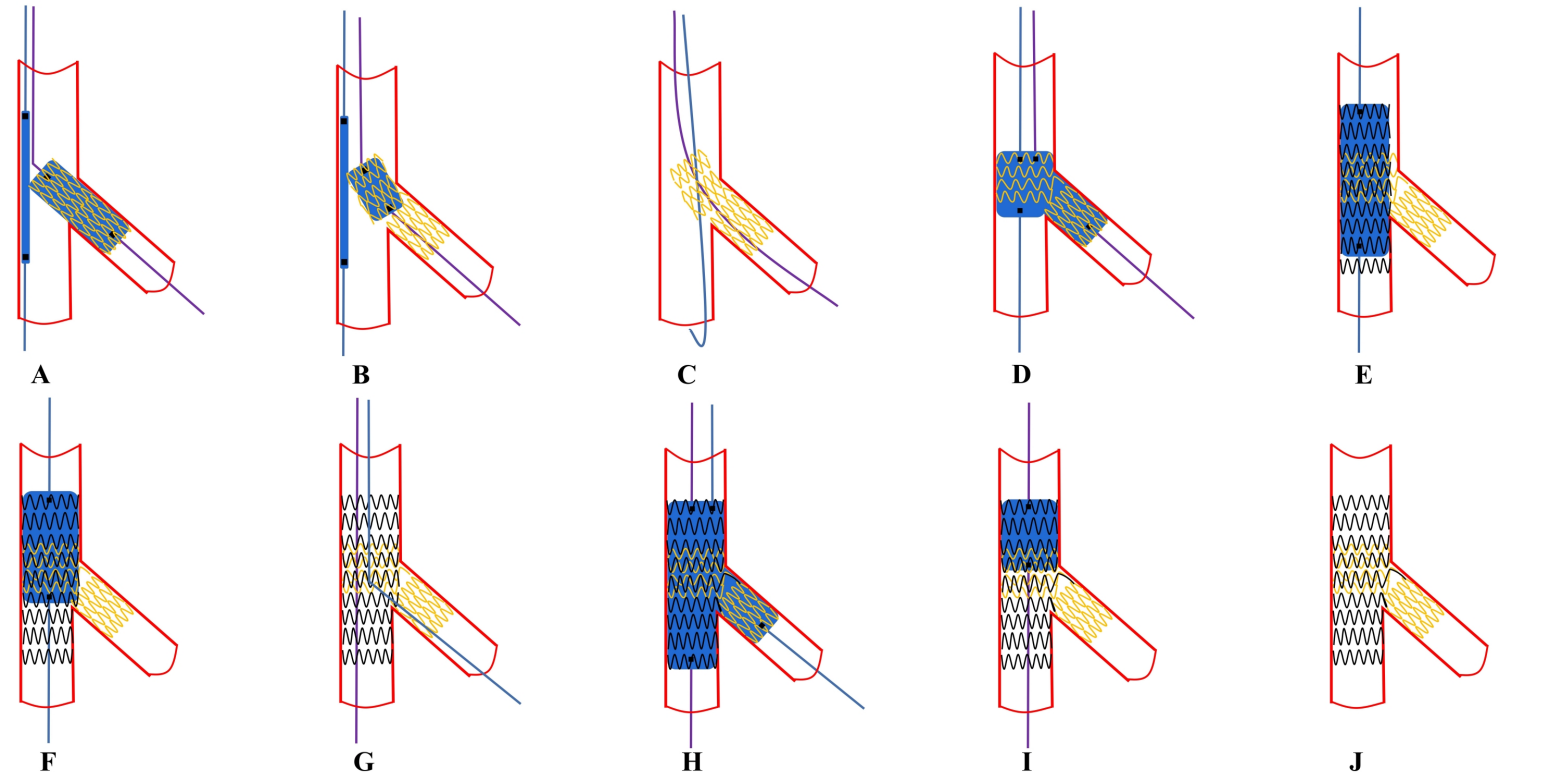
**Recommended steps of DK-Culotte stenting**. (A) Both the main vessel (MV) and side branch (SB) are wired, and then SB stent (sized 1:1 according to SB) is implanted in the SB protruding inside the proximal MV for 2–4 mm while the MV balloon is kept un-inflated into the MV. (B,C) After SB stent deployment, Initial proximal optimization technique (POT) at the level of proximal MV up to the carina level fully open the stent cell opening and then facilitate the SB guidewire cross the distal stent cell opening to the distal MV (the balloon inside the MV is still kept uninflated during this phase). (D) First kissing balloon inflation is performed to optimize the adherence, expansion, and morphology of the proximal segment of the stent. (E) A second stent is deployed through the opened SB strut cell with the diameter 1:1 according to the distal MV size. (F) Repeat POT with balloon sized 1:1 to proximal MV adjacent to the carina level. (G,H) Distal SB rewiring and the second kissing balloon inflation are performed. (I) The third POT is performed to decrease oval deformation of proximal stent. (J) Final effects are examined.

Two-stent techniques are pivotal in treating coronary bifurcation lesions, with various methods available to interventional cardiologists. Among these, some of the more commonly used two-stent techniques include Culotte, Mini-Culotte, DK-Culotte, Dk Mini-Culotte, Crush, Mini-Crush, DK-Crush, DK Mini-Crush, T-stent, and TAP. The less commonly utilized techniques include V-stent, SKS (simultaneous kissing stent), and Skirt techniques. The DK-Culotte and DK-Crush have replaced the traditional approaches because of their good morphological performance in *ex vivo* models [90, 91, 92] and validation in clinical studies [86]. *In vitro* experiments with DK Mini-Culotte and DK Mini-Crush techniques have been shown to provide superior morphological characteristics when compared to DK-Culotte and DK-Crush [93]. However, these findings await further validation from clinical studies. The DK-Culotte procedure is shown in Fig. 8. The key points of DK-Culotte include: two rounds of KBI, three POT, and the penetration of the distal strut cell. The main difference from the traditional Culotte procedure is that, after the guidewire passes through the SB strut cell into the distal part of MV, an KBI is performed to fully open the strut cell. Compared to the traditional Culotte procedure, the DK-Culotte technique avoids the “strangulation” of the MV stent by the SB struts, which results in the “napkin ring” effect [94]. This approach enables better stent apposition and expansion. *In vitro* studies suggest that the DK-Culotte technique is superior to both the conventional Culotte procedure and the prevalent DK-Crush technique [90]. After implantation of the SB stent, the first POT, aids in moving the guidewire into the distal strut cell of the MV, ensuring its complete opening. Following this, the deployment of the stent in the MV necessitates the second POT. This step plays a key role in facilitating the second rewiring through the distal strut cell of the MV stent, while simultaneously addressing the alignment of the proximal segment. Both POTs require the distal end of the POT balloon to be adjacent to the level of the carina. After the second KBI, a final POT is completed to correct the oval deformation of the proximal stent, expanding the proximal fragment from the SB takeoff. Additionally, close attention should be paid to the pre-embedding of the MV balloon prior to the release of the SB stent, which is crucial to the procedural success and requires careful execution.

**Fig. 9.**
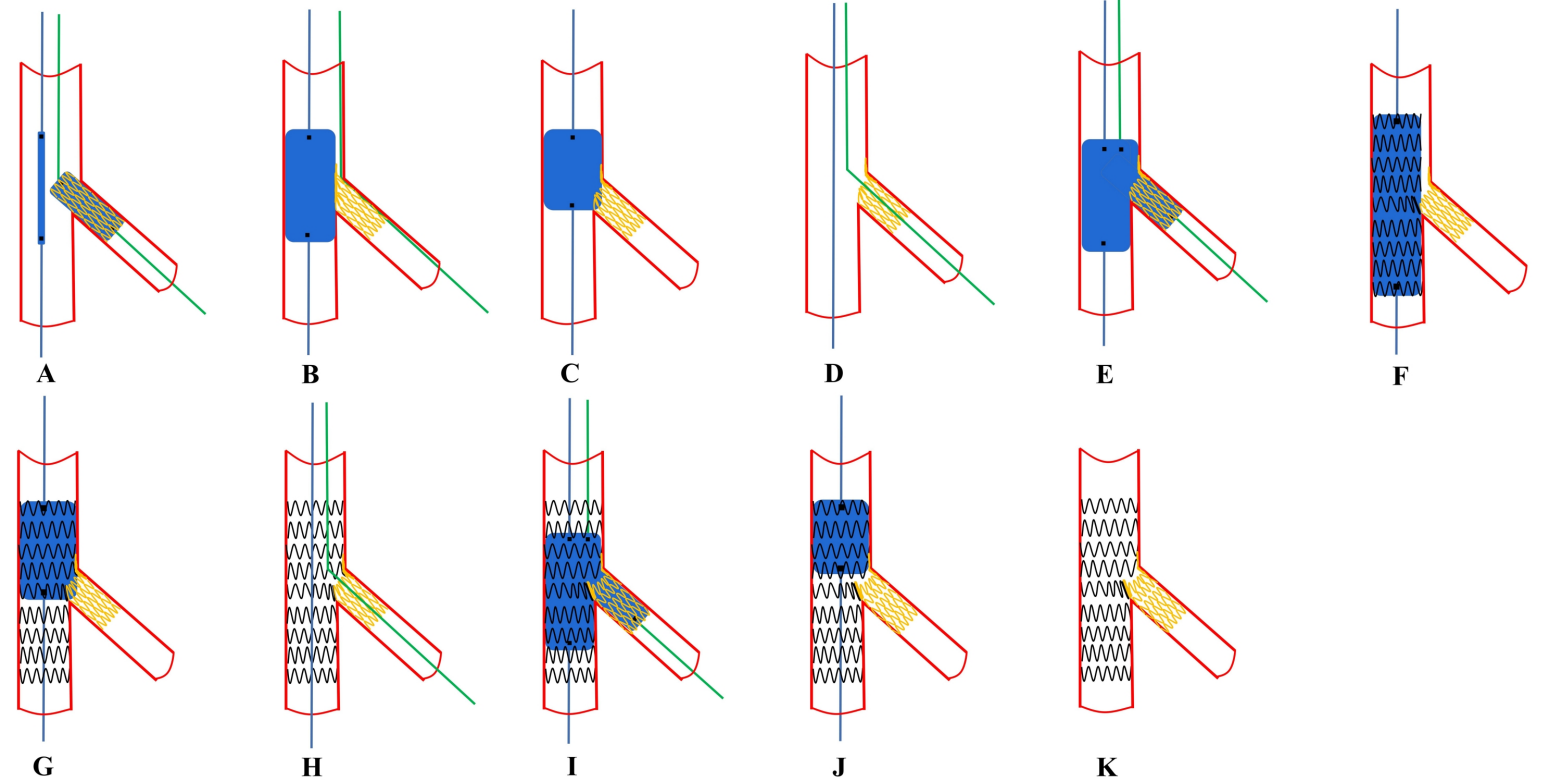
**Recommending steps of DK-Crush stenting**. (A) Both the main vessel (MV) and side branch (SB) are wired, and then SB stent (sized 1:1 according to SB) is implanted in the SB protruding inside the proximal MV for 2–4 mm while the MV balloon is kept un-inflated into the MV. (B) Balloon crush. After removal of the SB stent’s balloon, the protruding struts of the SB stent are crushed by the inflation of the balloon inside the MV sized to the distal MV (dMV). (C) Initial POT crush with a short balloon sized 1:1 to the proximal MV (pMV) to warrant optimal crushing without pMV malapposition. (D,E) proximal or non-distal SB rewiring and first kissing balloon inflation using two non-compliant balloons sized 1:1 according to the SB and dMV diameters. (F) MV stent is deployed after SB guidewire removal, stent implantation across the SB take-off with a stent diameter selected 1:1 according to the dMV size is performed. (G) Repeat POT with a balloon sized 1:1 to the pMV with meticulous attention paid to POT balloon position. (H) The SB is rewired crossing the SB ostium through a central or non-distal cell. (I) The second kissing balloon inflation is systematically needed (using short non-compliant balloons). (J) Final POT is performed with a short balloon sized 1:1 to the pMV. (K) Final effects are examined. POT, proximal optimization technique.

The DK-Crush procedure is shown in Fig. 9. The DK-Crush procedure, developed by Chen *et al*. [95], is an improvement over the traditional Crush technique. It involves one crush, two Sb rewires, two KBIs, and two POTs. After the compression of the SB stent struts against the MV wall, a guidewire is maneuvered through a non-distal strut cell of the SB stent to execute the initial KBI. This involves guiding the guidewire through the strut cells of the MV, then into the SB strut cell, and finally into the SB itself, setting the stage for the second KBI. Following the implantation of the MV stent, the first POT is performed. The final POT is performed after the final KBI. The final POT position is similar to that in the Culotte procedure. A series of landmark RCTs on the DK-crush technique have demonstrated the good efficacy of DK-Crush. In the European Clinical Guidelines on Haematopoietic Reconstruction, DK-Crush is a Class IIb recommendation [96]. The 16th EBC consensus also recommends it as the preferred two-stent technique for managing complex bifurcation lesions. However, it also emphasizes the high complexity of DK-Crush, which depends highly on the operators’ experience.

The T-stent and TAP procedures have been previously described in detail and will not be discussed here. The V-stent, SKS-stent, and Skirt procedures are not specifically recommended in the Consensus because of their use in specific bifurcation lesions and the high risk of restenosis and thrombosis.

## 7. Treatment Strategies for Left Main Bifurcation Lesions

Coronary angiography has revealed that the incidence of LM stenosis is 5%–7% [97, 98], with more than 80% involving the LM bifurcation [99]. Coronary artery bypass grafting (CABG) has traditionally been the primary choice for treating LM artery lesions and can reduce mortality by up to 65%. While PCI is an alternative non-invasive technique, there are still complication risks, including sudden death and stroke [100]. With the development of DES, intravascular imaging, optimization of antiplatelet therapy, and improvements in operator expertise, PCI is becoming a feasible alternative of CABG. A series of clinical trials [101, 102, 103, 104, 105, 106, 107, 108, 109, 110] demonstrated that PCI for elective unprotected LM artery lesions is safe and effective.

The optimal interventional treatment for LM bifurcation lesions remains a topic of debate, with results from several recent large RCTs presenting divergent findings. The 3-year outcomes of the DKCRUSH V study [111] demonstrated that the DK-Crush reduced the incidence of TLF, target vessel re-infarction, and in-stent thrombosis at 3 years compared to the PS procedure. The DEFINITION II trial demonstrated that systematic two-stent implantation was associated with better clinical outcomes than temporary stents for complex LM bifurcation lesions. However, the latest EBC Main trial showed that for patients with high-risk complex LM stem bifurcation lesions (Medina 1,1,1 or Medina 0,1,1), there was no significant difference between PS and dual stent implantation in terms of the primary endpoint (a composite of all-cause death, MI, and TLR at 12 months) at 1- and 3-year follow-up. Moreover, the incidence of TLR was lower in the PS group than in the dual stent group for the secondary endpoint (8% vs 14%, *p* = 0.02). These discrepancies may be derived from different study designs. The severity of disease in the EBC Main trial was relatively mild compared to the DKCRUSH V and DEFINITION II trials. Moreover, 53% of the dual stenting procedures applied in the EBC Main trial employed Culotte, and 33% utilized T-stenting versus TAP, whereas DK-crush was used in 77.8% of the double stenting procedures applied in the other two trials. Results of the EBC Main trial at 1 and 3 years showed the advantage of PS in simple bifurcation lesions. However, approximately 30% of patients present with complex bifurcation lesions for whom DK-Crush may be more suitable. This is consistent with the Chinese intervention guidelines for LM bifurcation lesions [112] and the EBC consensus [56]. These sources recommended classifying LM CBL according to the DEFINITION criteria. For simpler bifurcation lesions, a stepwise PS strategy is recommended. However, for complex LM CBL, a planned two-stent procedure, specifically the DK-Crush method, is advised. It’s important to note that the SBs of LM CBL are often critical, supplying significant myocardial territories, which necessitates careful consideration in the choice of interventional strategy.

Although double stenting leads to more MACE, the higher MACE rate was mainly driven by TLR. The TLR could be easily achieved with the application of drug coated balloons, cutting balloons, shockwave coronary intravascular lithotripsy (IVL), and excimer laser coronary atherectomy. In most cases, it is better to obtain higher procedure success at the slight risk of TLR in the treatment of complex LM bifurcation lesions. Therefore, the operators’ experience and team collaboration are critical to achieve procedure success.

## 8. Drug Coated Balloons

A promising alternative to stents, DCBs have emerged as an option for coronary in-stent stenosis, small vessel lesions, and bifurcation lesions. Evidence for DCB as a treatment option for *de novo* lesions has been mounting, particularly for small vascular lesions with a diameter of ≤2.75 mm, where clinical results are non-inferior to those achieved with stents [113]. Furthermore, DCB for *de novo* lesions has become a recommended clinical option as it avoids the use of stents with its corresponding drawbacks, such as prolonged dual antiplatelet therapy and revascularization challenges [58]. Current expert consensus suggests that DCB can be used in CBL, although the clinical evidence is still limited. When used in bifurcation lesions, there are two primary types of DCB, including the use of drug-eluting stents in MV with DCB in SB or using DCB in both MV and SB [114, 115, 116].

For most CBLs, the SB diameter is ≤2.75 mm, leading to an increasing number of a hybrid treatment strategies that involve using SB DCB and MV stents to manage true CBL. The HYPER study [117], which was recently published, treated 50 patients with true CBL using this approach. The study achieved a procedural success rate of 96%. During the 1-year follow-up, there was one perioperative myocardial infarction and one TLF in the segment treated with DES. The findings suggest that this hybrid strategy may be a safe and effective option for the treatment of true CBL, but further studies with controlled trials of standard treatment strategies are needed. The BEYOND study [118] was a prospective, multicenter, RCT designed to investigate the benefits of DCB compared to conventional balloon angioplasty in treating non-LM CBL. The study showed that DCB was superior to conventional balloons in terms of reducing lumen diameter stenosis and late lumen loss at 9-month follow-up. Moreover, it included patients with true CBL, with 33% of SB diameters measuring less than 2.0 mm and 31% of patients with diabetes mellitus. Hence, DCB is expected to yield better long-term outcomes in patients with small vessel disease.

For CBL >2.75 mm in diameter, primarily in LM CBL, the combination of DCB and stents remains an area with insufficient clinical evidence. Small prospective [119] and retrospective studies [120] have shown that SB-directed coronary atheromatous plaque resection of LM CBL, followed by DCB treatment, may reduce the number of stents and avoid complex stenting of major CBLs; these results demonstrated acceptable short-term efficacy compared to standard temporary SB stenting strategies. However, the limited sample size raises the need for larger clinical trials to validate the efficacy of treating LM CBL with DCB. Additional clinical studies on DCB for in-stent restenosis in LM CBL have been conducted [121]. In cases where the LCX diameter is ≤2.75 mm, the interventionalist should assess the individual risks and benefits to develop individualized strategies based on the patient’s risk of bleeding, the effectiveness of SB pre-treatment, and the difficulty of passing the SB guidewire through the SB ostium cell.

## 9. Conclusions

This review aims to bring interventionalists up-to-date on the latest CBL-related expert consensus, guidelines, and new and crucial research in this field. Coronary intracoronary imaging-guided CBL interventions can lead to better clinical outcomes, while DCB seems to be a promising approach for treating CBL, though additional large RCTs are necessary to validate and advance this technology. PS is considered the preferred strategy for most CBL cases, while planned dual-stenting provides superior benefits over PS for patients with complex CBL, as defined by the DEFINITION criteria. DK-Crush and DK-Culotte techniques are capable of achieving high success rates and favorable clinical outcomes. For successful bifurcation interventions, it is imperative that they are performed by experienced operators who possess proficiency in both general remedial procedures and planned dual stent implantations. Such expertise is crucial for navigating the intricate anatomical and procedural complexities associated with CBL, ensuring optimal patient outcomes.

## Abbreviations

PCI, percutaneous coronary intervention; CBLs, coronary bifurcation lesions; DCB, drug-coated balloon; EBC, European Bifurcation Club; SB, side branch; pMV, proximal main vessel; dMV, distal main vessel; 3D, three-dimensional; QCA, quantitative coronary analysis; FFR, fractional flow reserve; iFR, instantaneous wave-free ratio; QFR, quantitative flow ratio; KBI, kissing balloon inflation; IVUS, intravascular ultrasound; OCT, optical coherence tomography; RCT, randomized controlled trial; MACE, major adverse cardiac events; NC, non-compliant; POT, proximal optimal technique; TLF, target lesion revascularization failure; TLR, target lesion revascularization; JWT, jailed wire technique; JBT, jailed balloon technique; TAP, T-stenting and the small protrusion technique; DES, drug-eluting stents.
